# Mission impossible? Spatial context relearning following a target relocation event depends on cue predictiveness

**DOI:** 10.3758/s13423-023-02328-9

**Published:** 2023-07-11

**Authors:** Thomas Geyer, Artyom Zinchenko, Werner Seitz, Merve Balik, Hermann J. Müller, Markus Conci

**Affiliations:** 1 Department of Psychology, Ludwig Maximilian University of Munich, Leopoldstraße 13, 80802 Munich, Germany; 2 Munich Center of Neurosciences–Brain & Mind, Munich, Germany; 3 NICUM–NeuroImaging Core Unit Munich, Munich, Germany

**Keywords:** Attention, Visual search, Contextual cueing, Context adaptation

## Abstract

Visual search for a target is faster when the spatial layout of distractors is repeatedly encountered, illustrating that statistical learning of contextual invariances facilitates attentional guidance (contextual cueing; Chun & Jiang, 1998, *Cognitive Psychology, 36,* 28–71). While contextual learning is usually relatively efficient, relocating the target to an unexpected location (within an otherwise unchanged search layout) typically abolishes contextual cueing and the benefits deriving from invariant contexts recover only slowly with extensive training (Zellin et al., 2014, *Psychonomic Bulletin & Review, 21*(4), 1073–1079). However, a recent study by Peterson et al. (2022, *Attention, Perception, & Psychophysics, 84*(2), 474–489) in fact reported rather strong adaptation of spatial contextual memories following target position changes, thus contrasting with prior work. Peterson et al. argued that previous studies may have been underpowered to detect a reliable recovery of contextual cueing after the change. However, their experiments also used a specific display design that frequently presented the targets at the same locations, which might reduce the predictability of the contextual cues thereby facilitating its flexible relearning (irrespective of statistical power). The current study was a (high-powered) replication of Peterson et al., taking into account both statistical power and target overlap in context-memory adaptation. We found reliable contextual cueing for the initial target location irrespective of whether the targets shared their location across multiple displays, or not. However, contextual adaptation following a target relocation event occurred only when target locations were shared. This suggests that cue predictability modulates contextual adaptation, over and above a possible (yet negligible) influence of statistical power.

## Introduction

Humans display an impressive ability to extract statistical regularities from their environments and subsequently use the acquired knowledge to make predictions about upcoming sensory events, thus increasing the efficiency of perceptual processing. Recent work has started to specify the behavioral and neural mechanisms underlying the allocation of attention based on acquired long-term memories (LTMs) of environmental regularities, and describe how statistical learning influences visual search and object recognition (e.g., Geyer et al., 2021; Goujon et al., 2015; Võ et al., 2019). Although LTM-based mechanisms have been incorporated in current theories of visual search, such as Guided Search (e.g., Wolfe, 2021), these accounts, so far, pay little heed to the flexibility, or lack of it, of search-guiding LTM representations. For example, if a searched-for target is encountered a few times at a fixed location within an invariant spatial arrangement of distractor items, observers can learn these spatial distractor-target relations, or ‘contexts’, and use them to guide search to the target location—an effect referred to as ‘contextual cueing’ (CC; Chun & Jiang, 1998). However, work exploring the adaptability of CC showed that this LTM-based search-guidance effect is also severely limited (e.g., Annac et al., 2017; Conci & Müller, 2012; Conci et al., 2011; Conci & Zellin, 2022; Makovski & Jiang, 2010; Zellin et al., 2011, 2013a, 2014; Zinchenko et al., 2019): While CC for a given display arrangement typically develops quite rapidly (some 4–5 display repetitions suffice), its beneficial effect is substantially reduced after a sudden, but consistent, change of the target location within an otherwise unchanged spatial layout of the display; and, following such target relocations, it takes massive amounts of training to reestablish CC for the new target position (in Zellin et al., 2014, CC for the relocated targets only reemerged after 80 repetitions of each repeated display arrangement after several days of training). The reason for this limitation may lie in cue automatization. That is, when a given repeated display arrangement is reencountered, the initially acquired context automatically triggers attentional orienting to the old target location (by the cues raising the attentional priority of this location)—causing a persistent, and hard-to-overcome, cost after a consistent target-location change. In line with this, Zinchenko et al. (2020) showed that initial contextual learning was associated with a specific, lateralized marker of the evoked response in the EEG, the N1pc, arising 80–180 milliseconds (ms) after display onset at parieto-occipital electrodes contralateral to the (initial) target location, which is typically assumed to index automatic attentional-priority signaling (e.g., Wascher & Beste, 2010). After the target-location change (from one hemifield to the other), behavioral cueing vanished and, related to the new target location, the N1pc was reversed in polarity. Zinchenko et al. took this reversal to reflect a persistent ‘misguidance’ signal towards the old target location, preventing contextual adaptation to the relocated target. Thus, once learnt, repeated layouts trigger attentional-priority signals from memory, that, after target relocation, interfere with contextual relearning.

Importantly, the lack of CC after target location changes is unlikely to result from restrictions in memory capacity (Jiang et al., 2005) or a general lack of flexibility in learning invariant contextual information (Brockmole & Henderson, 2006; Brockmole & Le-Hoa Võ, 2010; Jiang & Wagner, 2004; Zang et al., 2017), which, in natural environments, may be supported in particular by the availability of additional semantic information (Goujon et al., 2012). Rather, the strong persistence of CC to update a given LTM representation appears to occur predominantly when the context remains the same and only the target changes—which is a situation that one frequently encounters in daily life (e.g., when searching for keys in an otherwise unchanged environment).

This view—that, once established, search-guiding memories are resistant to incorporating systematic target-location changes—has been challenged recently by Peterson et al. (2022). They proposed that CC is in principle flexible and open to adaptation, but previous studies that failed to find evidence for ready contextual relearning (following successful initial learning) suffered from sample sizes too small to reveal such adaptation effects. Increasing the number of participants tested to around 50 (from the more typical ~15 observers in, e.g., Zellin et al., 2014, or Zinchenko et al., 2020), Peterson et al. found not only that CC was successfully acquired during initial learning, but was also reestablished after only a few repetitions subsequent to a consistent target-location change. Accordingly, it would appear that larger sample sizes, compared with those used in previous studies, are required to demonstrate the ready recovery of cueing after target relocation and thus the inherent flexibility of acquired search-guiding LTM representations.

Of note, however, Peterson et al.’s (2022) experiments differed from previous studies not only in the sample size, but also procedurally with regard to how the presented search displays were constructed. In particular, in most of the previous studies (e.g., Zellin et al., 2014), displays—typically composed of a *T*-shaped target and some 11 *L*-shaped distractors—were distributed over a relatively large (underlying) spatial grid, such that each repeated and nonrepeated display arrangement would be paired with a unique target location. In Peterson et al. (2022), by comparison, the display grid was much smaller. As a result, search items (and in particular the target) would frequently share their spatial positions, across individual repeated (and nonrepeated) display arrangements. While these differences may appear negligible at first, they potentially constitute an important factor impacting the flexibility of contextual learning. For instance, when multiple repeated and nonrepeated displays are paired with the same target location (as in Peterson et al., 2022), the potential of these locations to become uniquely associated with a specific repeated distractor context would be diminished. In other words, there would be a reduction of cue predictiveness, which would weaken any association that may be formed between a given target location and repeated context. Conversely, however, weaker associations might facilitate the updating of the associative LTM representations subsequent to a target-location change (see, e.g., Wang et al., 2020; Zellin et al., 2013b, for related findings). Thus, in Peterson et al. (2022), the shared target positions across repeated and nonrepeated displays and its attendant effect on cue predictiveness may have facilitated contextual adaptation—over and above any benefits from increased statistical power for demonstrating successful contextual adaptation.^1^ Consistent with this possibility, it has been shown that learning of basic target–distractor associations takes into account the overall frequencies, or conditional probabilities, of the sensory events encountered, such as the ratio of repeated to nonrepeated displays (Zinchenko et al., 2018). Accordingly, contextual cueing may be considered as an ‘active-perception’ mechanism (Sauseng et al., 2015, Zinchenko et al., 2019), which also incorporates conditional probabilities of the location of searched-for targets in (sets of) repeated and nonrepeated distractor arrangements.

## Method

**Fig. 1:**
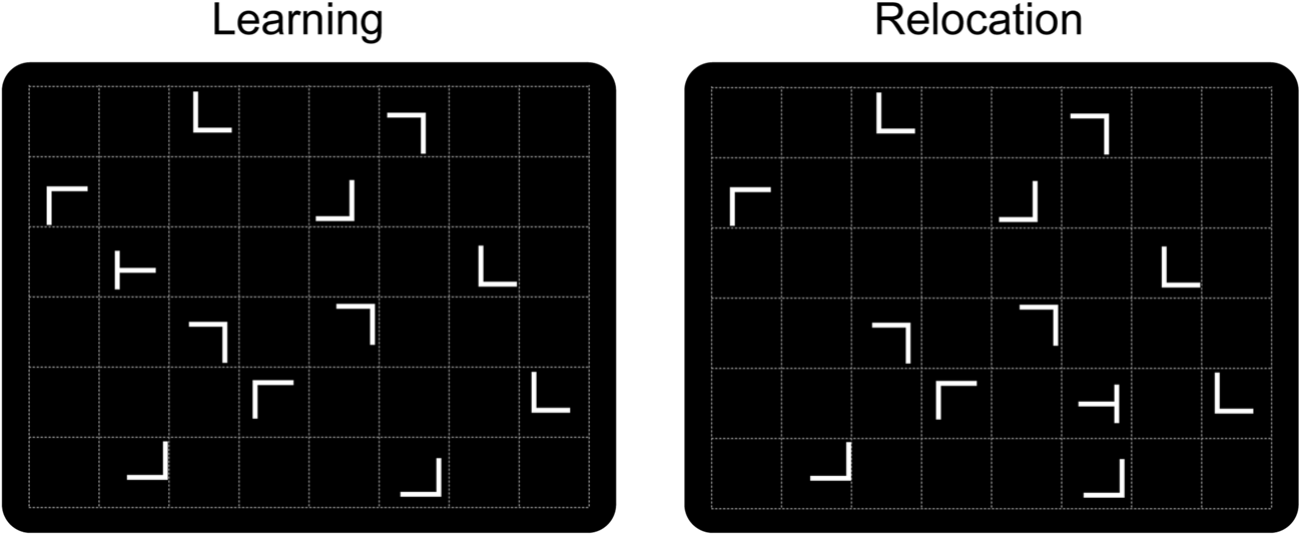
Example of a repeated-context search display in the learning (left) and relocation (right) phases of the experiment. Each display was presented with an initial target location during the learning phase; in the subsequent relocation phase, the target would then be presented at a new, previously empty position within an otherwise constant distractor layout. Note that the grey dotted lines, illustrating the 6 × 8 grid within which the display items were placed, were not shown in the actual search displays

The current study used a learning-/test-phase design to re-investigate the flexibility of CC acquired in the initial learning phase for incorporating a changed target position within an established distractor representation in the subsequent relocation phase. Following Peterson et al.’s (2022) suggestions concerning sample size, we recruited two large groups of observers (each comprising ~50 participants), while systematically comparing how more versus less overlapping target locations between repeated and nonrepeated displays would impact contextual adaptation. Each search display consisted of 12 items (one target and 11 distractors) that were arranged in an invisible grid of 6 horizontal rows × 8 vertical columns, that is, 48 possible item locations overall (see Fig. 1)—comparable to previous studies in which CC turned out to be substantially reduced following a target-location change (e.g., Zellin et al., 2013a, 2014). The basic procedure used in these previous studies was also adopted in the current “*nonshared*” group, where participants would be presented with a unique target location in each repeated and nonrepeated display in both the learning and relocation phases. By contrast, in the “*shared*” group, participants were presented with the very same search display configurations, except that a given target location would always be paired with two (randomly selected) search displays—namely, one repeated and one nonrepeated display. As a result, there were 12 distinct target locations in each of the two experimental phases, thus yielding 24 unique target locations overall. Accordingly, the predictiveness of the contextual cue was lower in this case because a given individual target location would be paired with a repeated context only on every second trial, on average (with a ratio of 1:2; in the nonshared condition, by contrast, the mapping between target locations and search displays was fully predictive, i.e., the display-to-target ratio was 1:1). Critically, the cue predictiveness in the shared group was fully comparable to Peterson et al. (2022). If statistical power is the only factor determining contextual adaptation, then both groups should display a reliable cueing effect following the target location change. However, if contextual adaptation is modulated by the spatial overlap of target locations, a recovery of CC after the target relocation should manifest only in the shared group—due to the low predictiveness of the contextual cues facilitating the updating of established context memories.

### Participants

The initial sample consisted of 100 participants, 50 of which were randomly assigned to the nonshared condition and 50 to the shared condition. We had to exclude six participants whose task performance was markedly (more than 2.5 *SD*s) worse than that of the other participants. Accordingly, the final sample consisted of 94 participants: 45 in the nonshared group and 49 in the shared group. These numbers are directly comparable to Peterson et al. (2022) who tested between 44 and 49 participants per experiment. The experimental procedure was in accordance with the guidelines of the Declaration of Helsinki and approved by the Ethics Committee of the Department of Psychology at LMU Munich. Informed consent was obtained from all participants prior to the experiment.

### Apparatus and stimuli

Example search displays are presented in Fig. 1. An array consisted of 12 grey (8.5 cd/m^2^) items, presented against a black background (0.02 cd/m^2^). One of the items was a *T*-shaped target rotated randomly by 90° to either the left or the right. The 11 remaining distractors were L-shapes rotated randomly in one of the four orthogonal orientations. All stimuli extended 0.7° × 0.7° of visual angle in width and height. Search displays were generated by placing the target and distractors randomly in the cells of a 6 x 8 matrix, with an individual cell size of 2.5° × 2.5°. Distractors were jittered horizontally and vertically in steps of 0.1°, within a range of ± 0.6°.

### Trial sequence

A trial started with the presentation of a fixation cross for 500 ms, followed by the onset of the search display. Participants were instructed to respond as quickly and accurately as possible to the orientation of the “T” (left vs. right). Each search display stayed on the screen until a response was issued. Participants responded to the left/right orientation the “T” target by pressing the left/right computer mouse button with their corresponding index finger. Following an erroneous response, a white minus sign appeared on the screen for 1,000 ms. Each trial was followed by a blank intertrial interval of 1,000 ms.

### Design

The experiment employed a mixed design with the within-subject factors Context (repeated, nonrepeated) and Epoch (1–10, with each epoch consisting of five consecutive trials blocks) and the between-subject factor Target position (nonshared, shared). Context had two levels: repeated and nonrepeated. A set of 12 repeated-context displays, each with an invariant distractor configuration, was generated for each observer and repeatedly presented throughout the experiment. Distractor orientations were also held constant across trials in repeated displays (cf. Chun & Jiang, 1998). For nonrepeated contexts, the placement (and orientation) of distractor items was generated randomly on each trial. The second factor, Epoch, divided the experiment into ten equally sized consecutive bins, with each bin/epoch consisting of 120 trials (averaging trials from five consecutive experimental blocks). The first four epochs of the experiment comprised a learning phase, where an initial set of (during these epochs) invariant target locations was paired with the repeated and nonrepeated distractor contexts. The subsequent epochs, epochs five to 10, consisted of a relocation phase in which the target was moved to a new, previously unoccupied, location in the search display; after this change, a given repeated distractor arrangement would be consistently re-presented together with the relocated target (see Fig. 1, for an example); the target-location change was also implemented in nonrepeated displays, only that the distractor arrangements continued to vary randomly across trials.

The two groups of observers were tested on two variants of the experiment, which varied in terms of their specific target-position conditions. In the “*nonshared*” group, 12 unique (and at the beginning of the search task randomly selected) target locations would be assigned to the 12 repeated search contexts in the learning phase, and 12 different target locations would be assigned to trials that presented nonrepeated search contexts. Two other sets of 2 × 12 target locations were used for repeated and, respectively, nonrepeated displays in the relocation phase. Thus, in the nonshared group, targets were presented at 48 distinct locations (within the 6 × 8 matrix) in the various (repeated/nonrepeated Context × learning/relocation Phase) conditions.^2^ By contrast, in the “*shared*” group, a given target location would always be paired with two (randomly selected) displays: one repeated and one nonrepeated. Accordingly, there were only 12 distinct target locations in the initial learning phase and a different set of 12 target locations in the subsequent relocation phase (thus, there were 24 distinct target locations used in the entire experiment). These measures ensured that the search displays were comparable in the two groups, except for the absolute number of target locations where a target could occur (48 vs. 24 in the nonshared and shared groups, respectively) and, associated with this, the consistency of the context-to-target mapping (1:1 vs. 1:2)—modulating the predictiveness of the contextual cues.

Each experiment started with a practice block of 24 randomly generated displays. All subsequent 50 experimental blocks consisted of 24 trials, 12 with repeated and 12 with nonrepeated context displays, presented in random order. After each block, observers continued with the experiment at their own pace. Overall, observers completed a total of 1,200 trials across the learning phase (Blocks 1–20 ~ Epochs 1–4) and the relocation phase (Blocks 21–50 ~ Epochs 5–10).

### Recognition test

After the end of the search task, participants were presented with a final recognition test that aimed to examine whether participants had explicit knowledge of the repeated contexts they had encountered in the search task they had just completed (see Chun & Jiang, 1998). The task was to distinguish between repeated and nonrepeated contexts via mouse-button responses. To this end, the 12 repeated-context displays and another 12 randomly generated nonrepeated contexts were presented in random order (24 trials in total). Repeated displays presented the target locations from the original learning phase (cf. Zellin et al., 2013a). The response was nonspeeded, and no error feedback was provided.

## Results

### Nonshared target locations

**Fig. 2:**
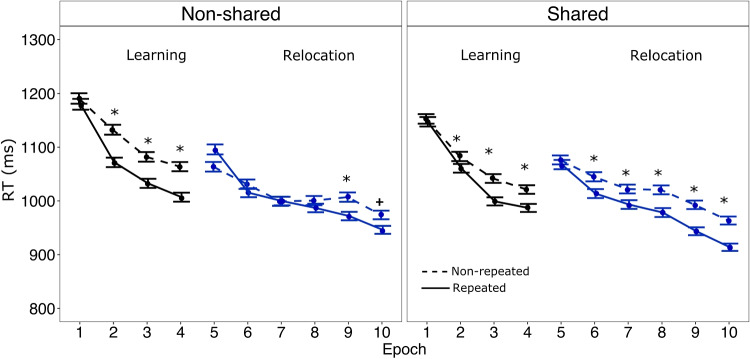
Mean reaction times (RTs, in ms with associated within-subjects standard errors from the ANOVA) for repeated and nonrepeated contexts across epochs in the nonshared (left) and shared (right) target position groups. Pairwise comparisons between RTs for repeated and nonrepeated contexts were computed for each epoch with stars denoting significant contextual-cueing effects (*p < .05; ^+^*p* < .10)

Individual mean error rates were calculated for each Context × Epoch combination. The overall error rate was low (1,65%) and a 2 × 10 repeated-measures ANOVA performed on the mean error rates with the within-subject factors Context (repeated, nonrepeated) and Epoch (1–10) revealed no significant main effects and/or interactions (all *p*s > .1). An analogous ANOVA carried out on the mean reaction times (RTs) after exclusion of incorrect responses and trial RTs exceeding ±3.0 standard deviations (3.76% of all trials), using the median absolute deviation (MAD) method (Leys et al., 2013) yielded significant main effects of Context, *F*(1, 44) = 5.31, *p* = .026, η_p_^2^ = 0.11, and Epoch, *F*(9, 396) = 43.25, *p* < .001, η_p_^2^ = 0.50. Targets were responded to faster overall when they appeared within repeated versus nonrepeated distractor contexts, with the overall CC-effect amounting to 26 ms. Also, search RTs became faster across epochs within a given phase: The mean RTs decreased by 150 ms and 123 ms across epochs in the learning and the relocation phase, respectively. Importantly, the Context × Epoch interaction turned out significant, *F*(9, 396) = 3.71, *p* < .001, η_p_^2^ = 0.08 (see Fig. 2, left panel), indicating that the change of the target location significantly impacted contextual learning.

To decompose this interaction, conditional, one-tailed *t* tests were performed that compared RTs between repeated and nonrepeated displays, starting at the 4th and 10th epochs followed by tests of the preceding epochs if revealing a significant difference (Peterson et al., 2022). These comparisons showed that CC emerged reliably in Epochs 2 (63 ms) to Epochs 4 (62 ms), smallest *t*-value = *t*(44) = −3.19, *p* < .01, Cohen’s *d* = 0.48. However, the cueing effect disappeared immediately after target relocation in epoch 5 (−27 ms) and did recover reliably only by Epoch 9 (32 ms), *t*(44) = −1.72, *p* = .045, *d* = 0.26. Note that we applied the step-wise analysis even though CC in Epoch 10 (28 ms) was only marginally significant, *t*(44) = −1.54, *p* = .065, *d* = 0.23.

### Shared target locations

Identical analyses were performed for the shared group (Fig. 2, right panel). The error rates were again low (1.21%) and not systematically affected by Context or Epoch (all *p*s > .1). A subsequent repeated-measures ANOVA conducted on the RTs revealed significant main effects of Context, *F*(1, 48) = 35.35, *p* < .001, η_p_^2^ = 0.42, and Epoch, *F*(9, 432) = 23.47, *p* < .001, η_p_^2^ = 0.33. Repeated contexts engendered faster search than nonrepeated contexts (mean CC-effect: 32 ms), and RTs again became faster across epochs within a given phase (mean RTs decreased by 149 ms and 135 ms across epochs in the learning and the relocation phase, respectively). Importantly, the Context × Epoch interaction was *not* significant, *F*(9, 432) = 1.58, *p* = .119, η_p_^2^ = 0.03—that is, CC was not impacted by the change of the target location. Conditional one-tailed *t* tests were again performed in reverse order, starting at Epochs 4 and 10 (Peterson et al., 2022). These comparisons revealed significant CC-effects in Epochs 2 (24 ms) to 4 (36 ms), smallest *t* value = *t*(48) = −1.83, *p* = 0.036, *d* = 0.26). During relocation, CC was reliable from Epoch 6 onwards (31 ms) until Epoch 10 (50 ms), smallest *t*-value = *t*(48) = −2.03, *p* = .023, *d* = 0.29).

### Combined analysis

To contrast CC between the two Target position groups, we carried out a mixed ANOVA with the within-subjects factors Context (repeated, nonrepeated) and Phase (learning, relocation), and the between-subjects factor Target position (shared, nonshared). This analysis yielded a significant main effect of Context, *F*(1, 92) = 27.57, *p* < .001, η_p_^2^’s = 0.23, comparable with the above-described analyses. Further, there was a reliable main effect of Phase, *F*(1, 92) = 42.73, *p* < .001, η_p_^2^’s = 0.32, indicating that RTs were overall slower in the learning phase compared with the relocation phase (1,077 and 1,004 ms, respectively). Importantly, there was a significant three-way interaction between Context, Phase, and Target position, *F*(9, 128)=1.98, *p* < .05, η_p_^2^ = 0.12, thus, showing between-group differences in CC across phases. Decomposing this interaction by means of post hoc tests revealed overall reliable CC-effects with both shared and nonshared targets (27 and 48 ms, respectively) in the initial learning phase, *t*s < 4.06, *p*s < 01, *d*s > .58. In the relocation phase, by contrast, CC was significant with shared target locations (36 ms), *t*(48) = −4.76, *p* < .001, *d* = 0.69, but nonsignificant with nonshared targets (10 ms), *t*(44) = −.65, *p* = .51, *d* = 0.10. This indicates once again that target overlap modulates the rate of contextual adaptation.^3^

### Recognition test

When comparing the hit rates (repeated displays correctly judged as repeated) against the corresponding false-alarm rates (nonrepeated displays incorrectly judged as repeated) by means of a Response type (hit, false alarm) × Target position (nonshared, shared) mixed-design ANOVA, we found that observers correctly recognized repeated displays in 51.8% of all trials, while falsely judging nonrepeated displays as repeated in 45.9% of trials, *F*(1, 90) = 7.85, *p* = .006, η_p_^2^ = 0.08 (main effect of response type). This suggests that participants were able to identify (at least some of) the repeated displays, which is consistent with previous findings that tested larger samples of observers (Geyer et al., 2020; Vadillo et al., 2016). However, participants’ explicit display knowledge was not influenced by target position overlap, as indicated by a nonsignificant Target position main effect, *F*(1, 90) = 0.19, *p* = .662, η_p_^2^ = 0, and a nonsignificant Response Type × Target Position interaction, *F*(1, 90) = 0.01, *p* = .937, η_p_^2^ = 0.

## Discussion

In line with Peterson et al.’s (2022) study of contextual adaptation, which tested a large sample size, the current experiment replicated a contextual facilitation effect—that is, faster RTs to repeated vs. nonrepeated displays—even after a sudden but consistent target-location change. However, carry-over of contextual facilitation across the change was observed only when targets from (paired) repeated and nonrepeated displays were presented with shared spatial locations, but not with nonshared target locations between the two types of display (thus, also replicating previous studies; e.g., Zellin et al., 2014). This pattern is difficult to square with accounts assuming that large sample sizes alone suffice to reveal efficient contextual adaptation. Instead, the results indicate that rather subtle, seemingly irrelevant details in the display design may engender marked differences in behavior. We suggest two (related) possibilities for explaining why contextual adaptation is more readily achieved with overlapping target locations.

First, carryover, or rapid recovery, of CC after target relocation would be expected if statistical learning is itself susceptible to contextual factors beyond the physical properties of the stimuli (Zinchenko et al., 2018), such as, in the present investigation, the specificity, or reliability, of target–context pairings. Accordingly, a highly predictive, 100% valid contextual cue (in the nonshared target condition), would give rise to the development of a reliable, yet relatively inflexible bias of search guidance compared with a contextual cue with lower predictiveness (i.e., 50% validity in the shared target condition). On this account, the flexibility of contextual cueing is a function of the predictiveness of the contextual cues with respect to the target location. However, according to our results, the influence of cue predictiveness becomes evident in particular after target relocation, that is, in “volatile” situations where an initial bias (towards the initial target location) competes with a to-be established novel bias (towards the changed target location).

Second, rather than being related to combined target-context learning, the increased flexibility of CC under overlap conditions might reflect other forms of statistical learning. One such form is probability cueing, that is, learning to expect targets to appear at likely (i.e., frequently repeated) versus less likely display locations (e.g., Geng & Behrmann, 2005). Another form is the learning of associations between repeated distractors on their own, without the distractor arrangement necessarily being linked to a specific target location; such leaning would provide a cue to locations that are unlikely to contain the target and so can be ignored (Vadillo et al., 2021), thus essentially revealing learning of the global context (of distractors; e.g., Brockmole et al., 2006) without a specific association to a given target. Thus, in the shared condition, it may be that the repeated target locations and the repeated distractor arrangements are learned relatively independently of each other, in the extreme without the formation of an association between the target location and the distractor context—owing to the target appearing at the same location in different display arrangements.

This idea receives some support from the pattern of RTs. As can be seen from Fig. 2, in the *shared* condition, the RTs show a marked slowing, by 70 ms, after target relocation, not only for repeated but also for nonrepeated contexts, *t*(48) = −3.30, *p* < .001. In contrast, in the *nonshared* condition, the RTs are essentially unchanged following target relocation, *t*(44) = 0.07, *p* = .93; in particular, there is no slowing for nonrepeated contexts. The RT performance with nonrepeated contexts may be considered to reflect probability cueing (without an additional influence from context repetition)—which is a major source of information in the condition with shared targets (given that the distractor arrangement has reduced information value in this condition). Accordingly, the sudden change of the previously learned target locations would render all initially acquired probability cues ‘invalid’, and this failure of probability cueing might transiently interfere with task performance on all trials (bringing about the general RT slowing), while target-location-independent context memory (of the distractor arrangement) would continue to facilitate search on trials with repeated contexts (accounting for the survival of the contextual-facilitation effect). Thus, in the shared condition, the pattern of RTs after the target-location changes might be explained by independent target-location and distractor-context learning, rather than in terms of more flexible target-context memories when the predictiveness of the context with respect to the target location is low.

However, our results are qualitatively different in the nonshared condition, in which we clearly replicated previous findings based on relatively small (yet in terms of power analyses justified) samples sizes that show a clear loss of CC (i.e., target–distractor relational LTM) after a consistent target-location change, given highly predictive contextual cues (for review see Geyer et al., 2021). This finding reinforces the proposal that when controlling, or essentially eliminating, overlap in target positions, adaptation of established contextual LTM representations to changed target locations is not observed even with large sample sizes—contrary to the statistical-power arguments of Peterson et al. (2022).

## Data Availability

Raw data are available at OSF (https://osf.io/nzuwk/?view_only=218f8f5791ae49f1b0f2b248a6353c59).

## Footnotes

1 Of note, it would theoretically be possible that a reduction of cue predictiveness conversely leads to more stable learning, with CC being particularly difficult to extinguish given variable cues (see, e.g., Turatto & Pascucci, 2016). However, this hypothesis is essentially opposite to the results revealed in previous, related findings (Peterson et al., 2022), and we therefore did not consider this option in further detail.

2 Although targets were randomly assigned to the display matrix, there is a potential (yet, not very likely) possibility that there might be some imbalance in the distribution of target locations across individual display quadrants. We tested this in two ways: first, by counting individual target positions for each participant and quadrant. These counts per quadrant were then analyzed by means of a 2 × 2 × 2 × 4 mixed-design ANOVA with the factors context, phase, target position, and display quadrant. This analysis revealed no main effects and no interactions (all *p*s > .24), thus demonstrating empirically that each quadrant was equally likely to contain targets in each experimental condition. In a second analysis, we identified the individual quadrants for each participant, which were most likely and second-most likely to contain a target. Of note, this procedure already demonstrated that the targets were distributed rather evenly across the quadrants (with an equal number of targets in 31% of all trials and a difference of just one or two targets across the two quadrants in another 61% of all trials). We then tested whether the difference in target counts between these two quadrants varied as a function of our experimental conditions. To this end, we computed another 2 × 2 × 2 mixed-design ANOVA with the factors Context, Phase and Target position on this target-quadrant difference measure, which again revealed no significant main effects and interactions (all *p*s > .05). This second analysis thus indicates that, in addition to the lack of an overall bias (as revealed by the results from the first analysis), individual variations in the placement of targets across specific quadrants did also not lead to a systematic spatial bias that might have influenced the pattern of results.

3 During initial learning, participants nevertheless displayed a numerically stronger CC-effect in the shared group compared with the nonshared group (see Fig. 2), which in turn may have impeded the (weak) recovery of CC in the relocation phase with nonshared targets. To test this, we selected the 20% of all participants in the non-shared group, which revealed the largest CC-effects during initial learning. In this subgroup, the mean cueing effect were 152 ms. In comparison, the 20% of all participants with the lowest CC-effects only revealed a mean cueing effect of −55 ms. However, the mean CC-effects in in these two subgroups of observers in the relocation phase were comparable (26 ms vs. 27 ms, respectively), *t*(29.88)= 0.042, *p* = .96, which suggests that the magnitude of CC in initial learning did not systematically influence the size of CC during relocation.
